# The Association of Perceived Vulnerability to Disease with Cognitive Restraint and Compensatory Behaviors

**DOI:** 10.3390/nu15010008

**Published:** 2022-12-20

**Authors:** Lindzey V. Hoover, Joshua M. Ackerman, Jenna R. Cummings, Ashley N. Gearhardt

**Affiliations:** Department of Psychology, University of Michigan, Ann Arbor, MI 48109, USA

**Keywords:** cognitive restraint, compensatory behaviors, perceived vulnerability to disease, fear of fat

## Abstract

Individual differences exist in perceived vulnerability to disease (PVD). PVD is associated with negative responses (e.g., disgust) towards individuals with obesity and heightened sensitivity regarding personal appearance. Through increasing fear of fat (FOF), PVD may be associated with cognitive restraint and compensatory behaviors. We utilized an adult sample (*n* = 247; 53.3% male sex assigned at birth) recruited through Amazon’s MTurk prior to the COVID-19 pandemic to investigate associations between PVD, cognitive restraint and compensatory behaviors. Participants completed the Perceived Vulnerability to Disease Scale, Eating Disorder Diagnostic Scale, Dutch Eating Behaviors Questionnaire, and Goldfarb’s Fear of Fat Scale. Mediation analyses were used to test our hypotheses. Perceived infectability (PVD-Infection) was associated with cognitive restraint and compensatory behaviors through increased FOF. Perceived germ aversion (PVD–Germ) was associated with cognitive restraint, but FOF did not mediate this association. Sex-stratified analyses revealed no significant sex differences. PVD may be an overlooked factor associated with cognitive restraint and compensatory behaviors in males and females. FOF was an important mediating factor in these associations. Increased engagement in cognitive restraint and compensatory behaviors may reflect attempts to reduce FOF. Future longitudinal research should explore whether PVD is a risk factor for cognitive restraint and compensatory behaviors.

## 1. Introduction

The coronavirus pandemic has brought the dangers of infectious disease to the forefront, but the dangers of pathogen transmission are not new. The near-constant threat of disease has fostered the development of defense mechanisms to protect our bodies from new and potentially deadly disease threats. The most well-known of these defenses is the immune system, which destroys pathogens detected within the body. However, humans also seem to have a second immune system known as the “behavioral immune system” [1,2,3] which, in contrast to the role of the reactive physiological immune system in combating internal pathogens, proactively prevents infection by facilitating detection of sensory cues (e.g., physical characteristics) in the environment that may indicate risk of disease exposure [1,2,3].

Perceived vulnerability to disease (PVD) is a trait-level indicator of behavioral immune activity that reflects both perceived infectability (PVD-Infection), a personal belief about susceptibility to infectious disease (e.g., if a disease is going around you will get it [4]) and germ aversion (PVD-Germ), the level of emotional discomfort experienced in situations where risk of disease transmission is high (e.g., someone sneezing without covering their mouth [4]). From an evolutionary perspective, PVD is highly beneficial to survival. Heightened sensitivity to environmental cues and higher perception of personal risk both encourage early detection and avoidance of potentially deadly pathogens before they can enter the body [3]. However, detection processes within the behavioral immune system are not perfect—because we cannot directly observe pathogens, these processes are sensitive to cues associated with disease but also can activate in response to cues that merely resemble or are heuristically associated with disease threat [3]. For example, physical characteristics (e.g., accident-related limb amputations), which are logically irrelevant to disease transmission, may activate this system and can result in negative responses toward the person associated with the cue [3,5].

### 1.1. Obesity as a Disease Cue

Obesity is a physical characteristic and chronic disease associated with disease-related perceptions [2,6,7]. Individuals with higher PVD are more likely to label individuals who appear heavier as obese [8] and are more prone to stigmatize individuals with higher body weight [6,8,9,10,11]. PVD has been directly associated with negative behavioral (e.g., avoidance) and cognitive (e.g., disgust) responses towards individuals with obesity [3]. PVD has also been associated with heightened sensitivity regarding one’s own appearance, including weight-related features [12]. Heightened attention to one’s own appearance combined with increased weight stigma may lead individuals with higher PVD to develop a fear of fat (FOF, i.e., avoidance of or aversion to fatness [13,14]) regarding their own body. Thus, heightened perceptions of vulnerability to disease could contribute to disordered eating behavior due to heightened FOF.

### 1.2. Fear of Fat and Disordered Eating

FOF is associated with cognitive restraint and compensatory behaviors [15,16,17]. Cognitive restraint refers to the desire to control or restrict food consumption (regardless of success [18,19]). Cognitive restraint is associated with FOF in clinical [20,21] and community samples [16,22,23,24,25]. FOF is also associated with restrictive eating disorders marked by compensatory behaviors (i.e., anorexia nervosa, bulimia nervosa [18,26,27,28]. Compensatory behaviors (e.g., fasting, abuse of laxatives/diuretics, vomiting, excessive exercising) are intended to counteract caloric intake to achieve a desired shape/weight or avoid fatness [18]. If PVD is also associated with FOF, then PVD may be a factor contributing to both cognitive restraint and compensatory behaviors.

### 1.3. The Current Study

We investigated the hypothesis that greater PVD is associated with cognitive restraint and compensatory behaviors through FOF in a community sample of 247 adults (age 21–70). This is (to our knowledge) the first study to investigate whether two dimensions of PVD (perceived germ aversion and perceived infectability) are associated with FOF, cognitive restraint and compensatory behaviors.

Female participants have been found to have higher PVD [4] and to be more prone to cognitive restraint and eating disorders than male participants [29]. Thus, we decided a priori to conduct exploratory sex-stratified analyses to investigate potential sex differences in the associations between PVD and FOF with cognitive restraint and compensatory behaviors.

## 2. Materials and Methods

### 2.1. Participants

We conducted secondary analyses using data from participants recruited on Amazon’s Mechanical Turk platform (MTurk) in 2019 prior to the COVID-19 pandemic. Participants completed questionnaires on their beliefs, behaviors, thoughts, and feelings related to eating and drinking (Qualifications: U.S. Location, HIT Approval Rate > 95%, Age > 18). Additional information (e.g., quality assurance steps) can be found in previously published work [30]. Original data have been shared on the Open Science Framework at https://osf.io/8kspw/, accessed on 10 August 2019.

All available data (*n* = 247, 53.3% male sex at birth) were included in the current study. The average age of participants was 36.8 years old (SD = 11.3, min–max = 21–70). The racial/ethnic distribution of the study was: 74.5% White, 15.4% Black, 6.9% Hispanic/Latinx, 4.5% Asian, 2.4% American Indian or Alaskan Native, and 0.8% other (percentages exceed 100% because participants could select one or multiple race/ethnicity). The sample overall was well-educated (12.1% high-school graduates, 18.2% some college, 9.7% associates degree, 47.4% bachelor’s degree, and 12.6% advanced degree (masters, Ph.D., M.D., J.D., etc.) Average participant body mass index (BMI) was “overweight” (M = 26.3, SD = 5.9, min–max = 17.7–55.8) with 3.1% of participants “underweight”, 42.3% “normal” weight, 36.6%, “overweight”, and 18.1% “obese”. Additional information about sample characteristics is presented in Table 1.

### 2.2. Procedures

Research procedures were approved by the University of Michigan Institution Review Board in accordance with provisions of the World Medical Association Declaration of Helsinki. Participants consented and completed questionnaires in randomized order through the MTurk platform. Participants were compensated USD 1.00 for their time (~25 min).

### 2.3. Measures

#### 2.3.1. Perceived Vulnerability to Disease Scale (PVD)

The Perceived Vulnerability to Disease Scale (PVD) [4] is a 15-item questionnaire that measures trait-level concerns about the transmission of infectious diseases. Participants rated each item on a 7-point Likert scale (1 = strongly disagree to 7 = strongly agree). The PVD questionnaire measures two factors: perceived germ aversion (PVD-Germ) and perceived infectability (PVD-Infection). Subscales were scored by averaging the items. The PVD-Germ subscale measures affective response to situations where pathogen transmission is likely (e.g., It really bothers me when people sneeze without covering their mouth, M = 4.4, SD = 1.1, min–max = 1.1–7.0, α = 0.76). The PVD-Infection Subscale assesses subjective beliefs about one’s own susceptibility to infectious disease as well as one’s personal beliefs about their immune functioning (e.g., In general, I am very susceptible to colds, flu and other infectious diseases, M = 3.4, SD = 1.2, min–max = 1.0–7.0, α = 0.82).

#### 2.3.2. Goldfarb Fear of Fat Scale (FOF)

The Goldfarb Fear of Fat Scale (FOF) [26] is a 10-item measure developed to differentiate normal versus abnormal FOF for early identification of patients at risk for bulimia nervosa. Participants rate how representative each statement is on a 4-point scale (1 = very untrue to 4 = very true). Questions include, “I am afraid to gain even a little weight”, and, “If I eat even a little, I may lose control and not stop eating”. The scale is scored by averaging all items (M = 2.4, SD = 0.9, min–max = 1.0–4.0, α = 0.92).

#### 2.3.3. Dutch Eating Behaviors Scale (DEBQ)

The Dutch Eating Behaviors Scale [19] is a 33-item self-report scale assessing restraint, emotional, and external eating behaviors. Participants answer questions about their eating behaviors on a 5-point scale (1 = Never to 5 = Always). Only the restraint subscale was included in analyses. Questions included on the restraint scale ask about frequency of engagement in cognitive restraint, such as “Do you try to eat less at mealtimes than you would like to eat?” The restraint subscale was calculated by averaging the 10 restraint items (M = 3.0, SD = 1.0, min–max = 1.0–5.0, α = 0.94).

#### 2.3.4. Eating Disorder Diagnostic Scale (EDDS)

The Eating Disorder Diagnostic Scale [31] is a brief, self-report measure of eating disorder diagnostic criteria (DSM–IV). Questions asked about the average number of occurrences of eating disorder symptoms per week over the past 3 months, for example, “How many times per week on average over the past 3 months have you made yourself vomit to prevent weight gain or counteract the effects of eating?” This scale assesses 4 types of compensatory behaviors (vomiting, laxatives/diuretics, excessive exercising, fasting) and possible scores range from 0 to 14 for each behavior. Endorsement was highest for fasting (M = 3.5, SD = 4.1, min–max = 0–14) followed by exercise (M = 2.9, SD = 4.1, min–max = 0–14), vomiting, (M = 2.3, SD = 4.2, min–max = 0–14) and laxative/diuretics (M = 2.2, SD = 3.9, min–max = 0–14). A compensatory eating behavior subscale was created by summing the total number of compensatory behaviors endorsed on these 4 questions (M = 10.8, SD = 15.6, min–max = 0.0–54.0, α = 0.95).

### 2.4. Data Analytic Plan

Analyses were conducted in IBM SPSS Statistics version 27, IBM Corp, Armonk, NY, USA [32]. Data were reviewed for normality, outliers (±3 SD), and missing values. Distributions met normality assumptions. Missing data were highest for BMI (*n* = 19). All other missing data ranged from *n* = 0 to *n* = 4. Missing data were removed using pairwise deletion. Thus, differences in *n* are a result of missing data.

Zero-order correlational analyses were conducted between demographic variables (age, sex, race/ethnicity, education, and BMI), cognitive restraint and compensatory behaviors to identify potential covariates (see Table A1). We created dummy codes for biological sex at birth (0 = male, 1 = female), race/ethnicity (0 = non-White, 1 = White), and education level (0 = associates or lower, 1 = bachelors or higher). Education was positively correlated with cognitive restraint (r = 0.20, *p* = 0.001) and compensatory behaviors (r = 0.29, *p* < 0.001). Including education in regression models for the relations of PVD subscales with cognitive restraint and compensatory behaviors did not alter significance (see Table A2). Thus, we report the unadjusted models.

Correlational analyses were conducted to investigate the hypothesized associations between PVD subscales, FOF, cognitive restraint and compensatory behaviors. Separate mediational analyses were conducted using the SPSS PROCESS Model 4 macro [33] to investigate whether FOF mediated the associations between PVD subscales, cognitive restraint, and compensatory behaviors. Both PVD subscales were included in the same model to account for shared variance between the subscales [4]. We used 10,000 bootstrap samples to create 95% bias-corrected confidence intervals to test the significance of indirect effects. Significance at *p* < 0.05 was indicated if the 95% confidence interval did not include zero. Sex-stratified mediation analyses were also conducted to investigate potential sex differences.

## 3. Results

### 3.1. Associations between PVD Subscales, Cognitive Restraint and Compensatory Behaviors

Table 2 presents zero-order correlations between PVD subscales, FOF, cognitive restraint and compensatory behaviors. PVD-Germ was positively associated with FOF (r = 0.15, *p* = 0.02) and cognitive restraint (r = 0.18, *p* = 0.005), but was not significantly associated with compensatory behaviors (r = 0.03, *p* = 0.66). PVD-Infection was positively associated with FOF (r = 0.45, *p* < 0.001), cognitive restraint (r = 0.21, *p* = 0.001) and compensatory behaviors (r = 0.35, *p* < 0.001).

### 3.2. Indirect Effects of PVD Subscales on Cognitive Restraint through FOF

**Figure 1:**
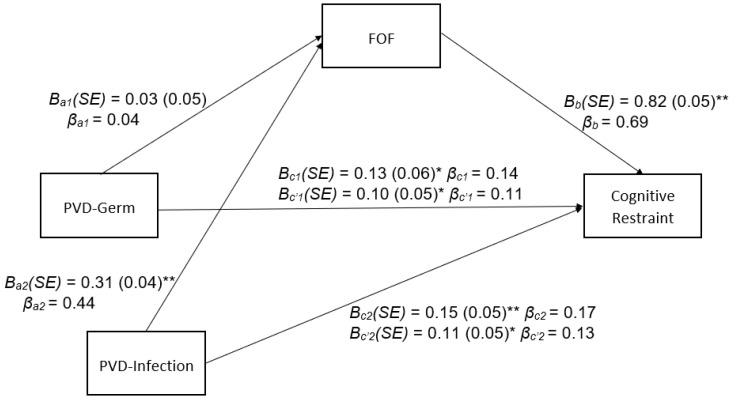
PVD Subscales and Cognitive Restraint Mediated by FOF. Process Model—4 path estimates from testing the indirect effect of perceived germ aversion (PVD-Germ) and perceived infectability (PVD-Infection) on cognitive restraint through fear of fat (FOF). Standardized coefficients (β), unstandardized coefficients (*B*), and standard errors (*SE*) are presented (* *p* < 0.05, ** *p* < 0.01).

Figure 1 presents results of mediational analyses for FOF as a potential mediator of the association between the PVD Subscales and cognitive restraint. The indirect effect of PVD-Infection on cognitive restraint through FOF was positive and significant, B (SE) = 0.26 (0.04), 95% CI [0.18, 0.34]. The direct effect of PVD-Infection on cognitive restraint was also significant. However, the indirect effect of PVD-Germ on cognitive restraint through FOF was not significant, B (SE) = 0.02 (0.04), 95% CI [−0.05, 0.09]. The direct effect of PVD-Germ on cognitive restraint was significant. Standardized coefficient betas suggest small-to-medium effect sizes of the associations between PVD subscales, FOF, and cognitive restraint.

### 3.3. Indirect Effects of PVD Subscales on Compensatory Behaviors through FOF

**Figure 2:**
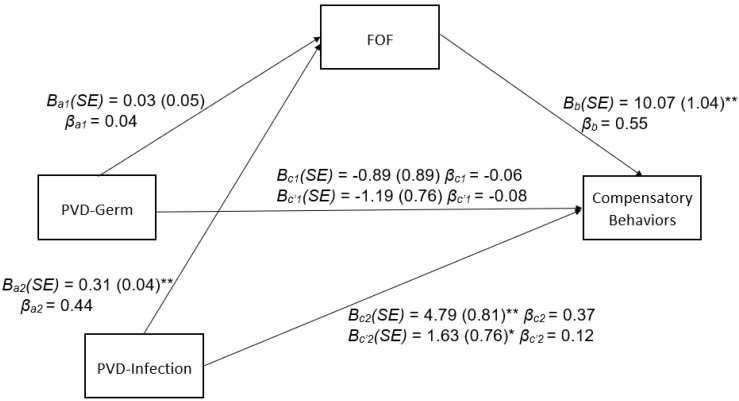
PVD Subscales and Compensatory Behaviors Mediated by FOF. Process Model—4 path estimates from testing the indirect effect of perceived germ aversion (PVD-Germ) and perceived infectability (PVD-Infection) on compensatory behaviors through fear of fat (FOF). Standardized coefficients (β), unstandardized coefficients (*B*) and standard errors (*SE*) are presented (* *p* < 0.05, ** *p* < 0.01).

Figure 2 presents results of mediational analyses for FOF as a potential mediator of the association between the PVD subscales and compensatory behaviors. The indirect effect of PVD-Infection on compensatory behaviors through FOF was significant, B (SE) = 3.16 (0.61), 95% CI [2.08, 4.47]. The direct effect of PVD-Infection on compensatory behaviors was also significant. In contrast, the indirect effect of PVD-Germ on compensatory behaviors through FOF was not significant, B (SE) = 0.30 (0.43), 95% CI [−0.55, 1.15]. The direct effect of PVD-Germ on compensatory behaviors was also not significant. Standardized coefficient betas suggest small-to-medium effect sizes of the associations between PVD subscales, FOF, and compensatory behaviors.

### 3.4. Sex-Stratified Analyses

**Figure A1:**
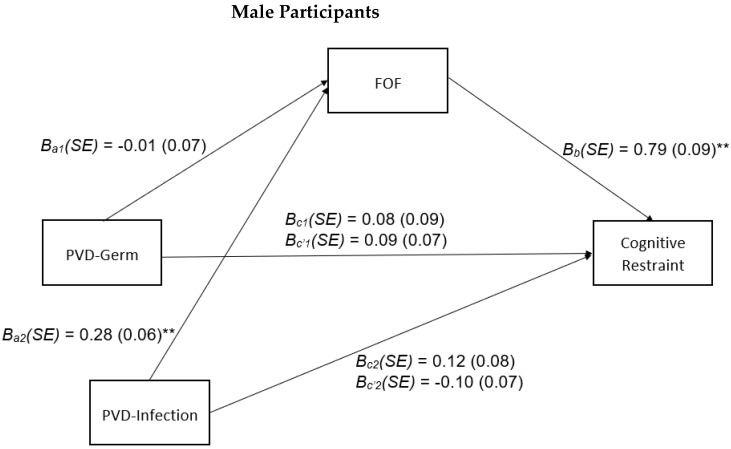

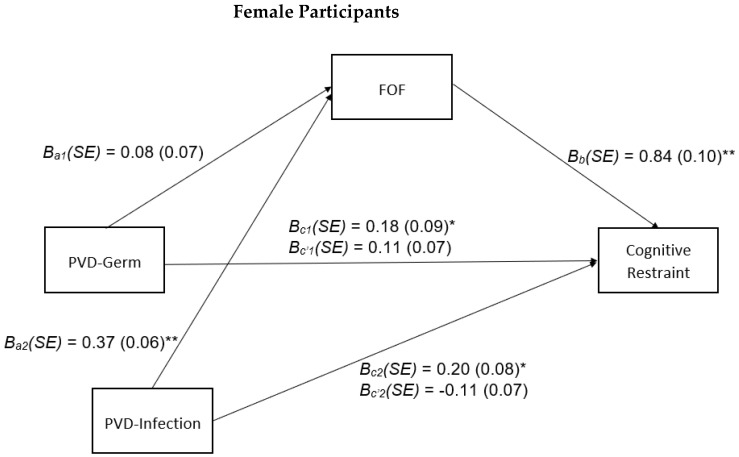
*Sex—Stratified Analyses for PVD Subscales, FOF, and Cognitive Restraint.* Process Model—4 path estimates from sex-stratified analyses testing the indirect effect of perceived germ aversion and perceived infectability on cognitive restraint through fear of fat. Unstandardized coefficients and standard errors are presented (* *p* < 0.05, ** *p* < 0.01). Indirect effect of PVD-Germ path for male, B (SE) = −0.02 (0.05), 95% CI [−0.11, 0.75], and female, B (SE) = 0.07 (0.06), 95% CI [−0.03, 0.19], participants. Indirect effect of PVD-Infection path for male, B (SE) = 0.22 (0.05), 95% CI [0.12, 0.34], and female, B (SE) = 0.31 (0.06), 95% CI [0.21, 0.45], participants.

**Figure A2:**
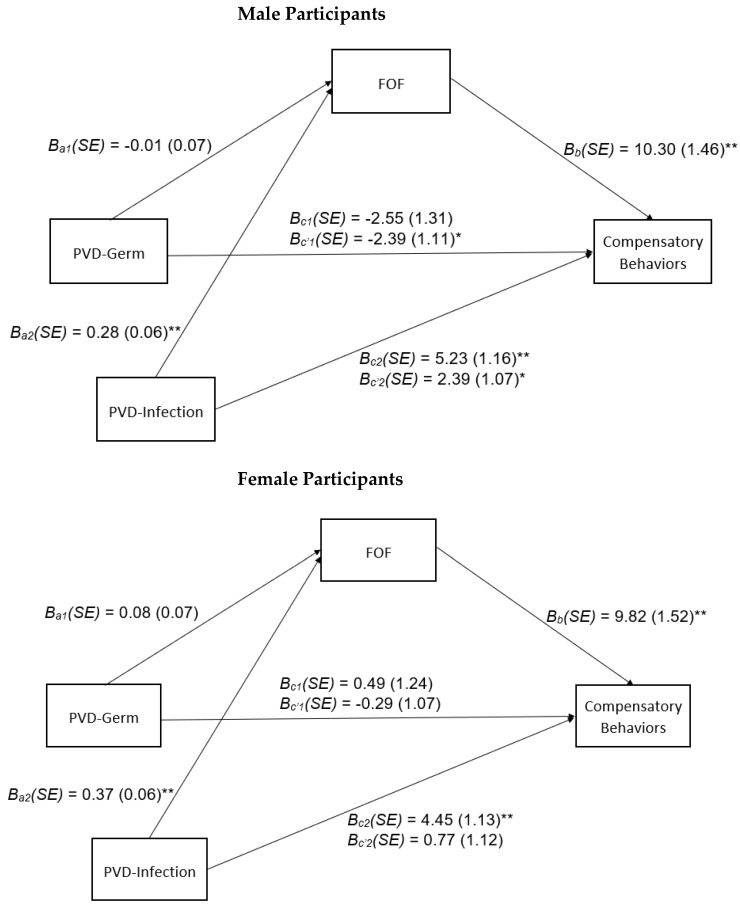
*Sex—Stratified Analyses for PVD-Infection, FOF, and Compensatory Behaviors*. Process Model—4 path estimates from sex-stratified analyses testing the indirect effect of perceived germ aversion and perceived infectability on compensatory behaviors through fear of fat. Unstandardized coefficients and standard errors are presented (* *p* < 0.05, ** *p* < 0.01). Indirect effect of PVD-Germ path for male, B (SE) = −0.15 (0.63), 95% CI [−1.53, 1.00] and female, B (SE) = 0.77 (0.68), 95% CI [−0.36, 2.35], participants. Indirect effect of PVD-Infection path for male, B (SE) = 2.84 (0.83), 95% CI [1.47, 4.73] and female, B (SE) = 3.68 (0.96), 95% CI [2.09, 5.88], participants.

Exploratory sex-stratified mediation analyses were conducted to investigate potential sex differences. Results indicated no significant sex differences between male and female participants. FOF did not significantly mediate associations between PVD-Germ and cognitive restraint or PVD-Germ and compensatory behaviors for male or female participants. FOF also remained a significant mediator of the associations between PVD-Infection and cognitive restraint and PVD-Infection and compensatory behaviors for both male and female participants (see Figure A1 and Figure A2).

## 4. Discussion

This is, to our knowledge, the first study to investigate the association between PVD, cognitive restraint, and compensatory behaviors. In a community sample of 247 adult participants, we found that perceived infectability was associated with cognitive restraint and compensatory behaviors and that FOF partially mediated these associations. Perceived germ aversion was significantly associated with cognitive restraint (but not compensatory behaviors). However, FOF did not significantly mediate the association between perceived germ aversion and cognitive restraint. Sex-stratified analyses revealed no significant sex differences between male and female participants. Perceived infectability may be an overlooked factor associated with cognitive restraint and compensatory behaviors in males and females through FOF. Implications of these findings are discussed below.

### 4.1. PVD, FOF, Cognitive Restraint, and Compensatory Behaviors

Perceived infectability refers to personal beliefs about susceptibility to infectious disease that stem, in large part, from one’s history of infections [4]. Results indicated that those with a heightened concern about their own susceptibility to disease endorsed both higher cognitive restraint and compensatory behaviors, which was partially explained by higher FOF. One possible explanation for these findings is that those higher in perceived infectability may perceive themselves as being more susceptible to obesity. This hypothesis aligns with past research indicating obesity is a physical condition that can be erroneously identified as an indicator of disease transmission by the behavioral immune system [2,6,7]. The misidentification of obesity as a disease cue in those high in perceived infectability could lead to an increased fear of the “disease” of fatness.

FOF mediation findings suggest possible future directions for reducing cognitive restraint and compensatory behaviors. Reducing FOF in individuals with high perceived infectability may be important for reducing disordered eating. For example, delivering interventions designed to reduce internalized weight stigma (e.g., cognitive-behavioral treatment to cope with internalized weight stigma [34]) to individuals with high perceived infectability may be useful in reducing cognitive restraint and compensatory behaviors. Illness during childhood is an important predictor of heightened perceived infectability in adults [35], so reducing FOF in children and adolescents who have experienced significant illness may also be useful in preventing the development of cognitive restraint and compensatory behaviors.

FOF partially mediated the associations between perceived infectability, cognitive restraint, and compensatory behaviors, leaving the possibility of other mediators. For instance, the associations between perceived infectability, cognitive restraint, and compensatory behaviors could reflect broader concerns about negative health outcomes associated with obesity. Perceived infectability is strongly associated with health anxiety [4], and it is plausible that those high in perceived infectability may engage in cognitive restraint and compensatory behaviors to minimize the risk of negative health outcomes associated with obesity.

Contrary to our hypothesis, perceived germ aversion was not related to compensatory behaviors. Although perceived germ aversion demonstrated a small, but significant association with cognitive restraint, FOF did not significantly mediate this association. Perceived germ aversion is thought to reflect emotional discomfort with situations, indicating an increased risk of disease transmission [4] and has been found to significantly overlap with pathogen disgust [9]. A growing body of research suggests that disgust-based avoidance is an important factor contributing to the development and maintenance of cognitive restraint and compensatory behaviors [36]. Future research should explore the role of disgust in the association between perceived germ aversion and cognitive restraint.

### 4.2. Sex-Stratified Analyses

Results of sex-stratified analyses reflected results in the full sample. FOF remained a significant mediator for associations between perceived infectability (but not perceived germ aversion), cognitive restraint, and compensatory behaviors in male and female participants. Males have historically been under researched in the context of disordered eating behaviors [37]. Our results suggest that evolutionary processes associated with disease threat could be one factor contributing to disordered eating for males and females, warranting further investigation.

### 4.3. Strengths and Limitations

The current study had several notable strengths. We had a relatively large sample size (*n* = 247) with a balanced sex distribution (53.3% male). The sample included a wide range of ages (M = 36.8, min–max = 21–70) and BMI (M = 26.28, min–max = 17.7–55.8). Study data included assessments of both cognitive restraint and compensatory behaviors and, to our knowledge, was the first to investigate their association with perceived disease vulnerability.

There are limitations of this study that should also be considered. Cognitive restraint and compensatory behaviors were assessed using self-report measures. Although these are validated scales [19,31], self-reported measures tend to over-represent disordered eating behavior [38,39,40]. Future research should utilize clinical interviews to assess cognitive restraint and compensatory behaviors. Data were collected using Amazon’s MTurk, which has historically raised some concerns about data quality. Recommended approaches (95% approval, 5000 HITS, attention check questions [41]) were implemented to minimize these concerns. Another important next step is to utilize clinical samples with disordered eating to explore associations between PVD, cognitive restraint, and compensatory behaviors. Although there was some diversity in this sample, it consisted predominantly of White participants (74.5%) and was underpowered to investigate differences in race/ethnicity. Future research should recruit a more diverse sample to allow for investigation of racial/ethnic differences in PVD, cognitive restraint, and compensatory behaviors. Factors other than FOF likely contribute to an association between PVD and disordered eating. Both PVD and disordered eating are associated with anxiety more broadly [42,43], disgust sensitivity [44,45,46], and obsessive-compulsive tendencies [43,44,46,47,48]. This dataset did not include measures on these constructs, but future research that investigates anxiety, disgust sensitivity, and OCD as potential mediators is an important future direction. While eating disorders and obesity are distinct constructs, they are not mutually exclusive of each other. Rates of eating disorders are elevated in individuals with obesity [49] and binge eating often occurs when restrictive eating fails [50,51]. While we did assess BMI as a potential covariate in this study, the study was not well suited to disentangle the complex associations between eating disorders and obesity. Future research should aim to investigate these complex associations. The study utilized a cross-sectional design, which does not allow for us to make causal inferences. Future research should utilize experimental or longitudinal designs that are better positioned to investigate causal inferences. For example, it may be possible to experimentally prime pathogen threat cues [52,53] to investigate whether being primed for PVD increases fear of fat, stigmatizing attitudes, or desire to engage in disordered eating behaviors. Assessing PVD in childhood would allow for follow-up during adolescence (a high-risk period for onset of eating disorders [54]) to see if childhood PVD is predictive of adolescent disordered eating behaviors. If PVD was predictive of future onset of disordered eating behaviors, it may be a useful screening tool for early intervention. Understanding the causal effect of disease salient environmental cues in increasing FOF and engagement in cognitive restraint and compensatory behaviors may be particularly important considering heightened media coverage surrounding disease transmission during the pandemic.

## 5. Conclusions

The role that evolved psychological mechanisms play in cognitive restraint and compensatory behaviors is an important and understudied area of psychological research. Our results indicate that people who perceive themselves to be more susceptible to infection (i.e., perceived infectability) are more likely to engage in cognitive restraint and compensatory behaviors. No differences were found between male and female participants, suggesting that perceived infectability might be a novel factor that can contribute to existing gaps in the understanding of disordered eating in males. Increased engagement in cognitive restraint and compensatory behaviors due to perceptions of obesity as a disease-relevant cue may be an attempt to reduce risk or fear of becoming fat. Future longitudinal research is needed to explore whether individual differences in perceived infectability is a risk factor for the development of cognitive restraint and compensatory behaviors.

Although data were collected before the coronavirus pandemic, consideration of how the pandemic might relate to these findings is particularly relevant. Increased salience of vulnerability to coronavirus combined with the specific identification of obesity as a risk factor for severe disease trajectory could have concerning implications for engagement in cognitive restraint and compensatory behaviors. Early research during the pandemic found that participants with a current or history of an eating disorder endorsed increased cognitive restraint and compensatory behaviors [55]. Such findings might be particularly likely in individuals with high PVD. Future research should explore the potential impact of the coronavirus pandemic on cognitive restraint and compensatory behaviors.

## Figures and Tables

**Table 1:** Demographic characteristics.

	*n* (%)
Age (M = 36.8, SD = 11.3, min–max = 21–70)	
21–29	73 (29.6%)
30–39	101 (40.8%)
40–49	33 (13.4%)
50–59	23 (9.3%)
60–69	16 (6.5%)
70	1 (0.4%)
Sex at Birth	
Male	131 (53.3%)
Female	115 (46.7%)
Racial Identity ^±^	
American Indian/Alaskan Native	6 (2.4%)
Hispanic/Latino	17 (6.9%)
Asian	11 (4.5%)
Black/African American	38 (15.4%)
White	184 (74.5%)
Other	2 (0.8%)
Education	
High school graduate	30 (12.1%)
Some college	45 (18.2%)
Associates degree	24 (9.7%)
Bachelor’s degree	117 (47.4%)
Advanced degree	31 (12.6%)
Income	
Less than USD 10,000	15 (6.1%)
USD 10,000–USD 19,999	18 (7.3%)
USD 20,000–USD 29,999	31 (12.7%)
USD 30,000–USD 39,999	45 (18.4%)
USD 40,000–USD 49,999	31 (12.7%)
USD 50,000–USD 59,999	25 (10.2%)
USD 60,000–USD 69,999	19 (7.8%)
USD 70,000–USD 79,999	20 (8.2%)
USD 80,000–USD 89,999	8 (3.3%)
USD 90,000–USD 99,999	10 (4.1%)
USD 100,000–USD 149,999	16 (6.5%)
More than USD 150,000	7 (2.9%)
Subjective Socioeconomic Status ^¥^	
1	3 (1.2%)
2	19 (7.7%)
3	32 (13.0%)
4	32 (13.0%)
5	60 (24.3%)
6	32 (13.0%)
7	35 (14.2%)
8	23 (9.3%)
9	9 (3.6%)
10	2 (0.8%)
BMI (M = 26.3, SD = 5.9, min–max = 17.7–55.8)	
Underweight (BMI < 18.5)	7 (3.1%)
Normal Weight (BMI 18.5–24.9)	96 (42.3%)
Overweight (BMI 25.0–29.9)	84 (36.6%)
Obese (BMI > 30)	41 (18.1%)

Notes: Differences in *n* are due to “prefer not to answer” responses. ^±^ Percentages for Race/Ethnicity exceed 100% because of the option to select multiple response options. ^¥^ Subjective Socioeconomic Status indicates participants self-ranking on a ladder representing people in the US with 10 = people who are best off (most money, most education, most respected jobs) and 1 = worst off (least money, least education, least respected jobs).

**Table 2:** Zero-Order Correlation Matrix PVD, FOF, Cognitive Restraint, and Compensatory Behaviors.

		PVD-Germ	PVD-Infection	FOF	Cognitive Restraint	Comp Behaviors
PVD–Germ	***r***		0.25 ***	0.15 *	0.18 **	0.03
	***n***		245	245	245	245
PVD–Infection	***r***			0.45 ***	0.21 **	0.35 ***
	***n***			245	245	245
FOF	***r***				0.65 ***	0.60 ***
	***n***				246	246
Cognitive Restraint	***r***					0.48 ***
	***n***					247
Comp Behaviors	***r***					
	***n***					

Notes: * indicates significance at *p* < 0.05. ** indicates significance at *p* < 0.01. *** indicates significance at *p* < 0.001. “FOF” indicates Goldberg’s Fear of Fat Scale. “PVD–Germ” indicates the Perceived Vulnerability to Disease Germ Subscale. “PVD–Infection” indicates the Perceived Vulnerability to Disease Infectability Subscale. “Cognitive Restraint” indicates the Dutch Eating Behavior Questionnaire Restraint Scale. “Comp Behaviors” indicates Eating Disorder Diagnostic Scale—Compensatory Behaviors Subscale.

## Data Availability

Original data have been shared on the Open Science Framework at https://osf.io/8kspw/, accessed on 10 August 2019. No new data were collected for this study.

## Appendix A

**Table A1:** Correlation matrix with potential covariates.

		PVD-Germ	PVD—Inf	FOF	Cog Restraint	Comp Behaviors	BMI	Age	Sex	Education	Race/Ethnicity
PVD—Germ	r		0.25 ***	0.15 *	0.18 *	0.03	−0.13	0.02	−0.22 **	−0.06	0.11
	*n*		245	245	245	245	226	245	244	245	245
PVD—Inf	r			0.45 ***	0.21 **	0.35 ***	0.06	0.09	−0.10	0.14 *	0.13 *
	*n*			245	245	245	226	245	244	245	245
FOF	r				0.65 ***	0.60 ***	0.07	<0.01	−0.03	0.19 **	≤0.01
	*n*				246	246	227	246	245	246	246
Cog Restraint	r					0.48 ***	0.01	0.02	−0.02	0.21 **	−0.04
	*n*					247	228	247	246	247	247
Comp Behaviors	r						−0.12	≤0.01	−0.07	0.29 ***	≤0.01
	*n*						228	247	246	247	247
BMI	r							−0.03	−0.05	−0.16 *	−0.03
	*n*							228	227	228	228
Age	r								0.18 **	0.09	0.19 **
	*n*								246	247	247
Sex	r									−0.10	0.16 *
	*n*									246	246
Education	r										−0.16 *
	*n*										247
Race/Ethnicity	r										
	*n*										

Notes: * indicates significance at *p* < 0.05. ** indicates significance at *p* < 0.01. *** indicates significance at *p* < 0.001. “FOF” indicates Goldberg’s Fear of Fat Scale. “PVD—Germ” indicates the Perceived Vulnerability to Disease Germ Subscale. “PVD—Inf” indicates the Perceived Vulnerability to Disease Infectability Subscale. “Cog Restraint” indicates the Dutch Eating Behavior Questionnaire Restraint Scale. “Comp Behaviors” indicates Eating Disorder Diagnostic Scale—Compensatory Behaviors Subscale. “BMI” indicates body mass index.

## Appendix B

**Table A2:** Regression analyses for potential covariates.

	*t*	*p*	*b (SE)*	β	*f*	*p*	*Adj. R* ^2^
Cog Restraint							
Overall model					9.51	<0.001	0.07
Constant	7.40	<0.001	2.03 (0.28)				
Education	3.29	0.001	0.42 (0.13)	0.20			
PVD—Germ	3.04	0.003	0.18 (0.06)	0.19			
Cog Restraint							
Overall model					8.86	<0.001	0.06
Constant	11.67	<0.001	2.31 (0.20)				
Education	2.68	0.01	0.35 (0.13)	0.17			
PVD—Infection	2.93	0.004	0.16 (0.05)	0.18			
Comp Behaviors							
Overall model					11.16	<0.001	0.08
Constant	0.62	0.54	2.58 (4.19)				
Education	4.70	<0.001	9.22 (1.96)	0.29			
PVD-Germ	0.71	0.48	0.63 (0.88)	0.04			
Comp Behaviors							
Overall model					26.72	<0.001	0.17
Constant	−2.77	0.01	−7.91 (2.85)				
Education	4.13	<0.001	7.72 (1.87)	0.24			
PVD-Infection	5.39	<0.001	4.15 (0.77)	0.32			

Notes: “PVD—Germ” indicates the Perceived Vulnerability to Disease Germ Subscale. “PVD—Infection” indicates the Perceived Vulnerability to Disease Infectability Subscale. “Cog Restraint” indicates the Dutch Eating Behavior Questionnaire Restraint Scale. “Comp Behaviors” indicates Eating Disorder Diagnostic Scale–Compensatory Behaviors Subscale.
